# Patients With Becker Muscular Dystrophy Have Severe Paraspinal Muscle Involvement

**DOI:** 10.3389/fneur.2021.613483

**Published:** 2021-05-21

**Authors:** Aisha M. Sheikh, Karen Rudolf, Josefine de Stricker Borch, Tahmina Khawajazada, Nanna Witting, John Vissing

**Affiliations:** Copenhagen Neuromuscular Center, Department of Neurology, Rigshospitalet, University of Copenhagen, Copenhagen, Denmark

**Keywords:** paraspinal muscles, Becker muscular dystrophy, quantitative muscle MRI, quantitative trunk strength, fat fraction

## Abstract

**Introduction:** Paraspinal muscles are important for gross motor functions. Impairment of these muscles can lead to poor postural control and ambulation difficulty. Little knowledge exists about the involvement of paraspinal muscles in Becker muscular dystrophy.

**Objective:** In this cross-sectional study, we investigated the involvement of paraspinal muscles with quantitative trunk strength measure and quantitative muscle MRI.

**Methods and Materials:** Eighteen patients with Becker muscular dystrophy underwent trunk, hip, and thigh strength assessment using a Biodex dynamometer and an MRI Dixon scan. Fourteen age- and body mass index-matched healthy men were included for comparison.

**Results:** Muscle fat fraction (FF) of the paraspinal muscles (multifidus and erector spinae) was higher in participants with Becker muscular dystrophy vs. healthy controls at all three examined spinal levels (C6, Th12, and L4/L5) (*p* < 0.05). There was a strong and inverse correlation between paraspinal muscle FF and trunk extension strength (ρ = −0.829, *p* < 0.001), gluteus maximus FF and hip extension strength (ρ = −0.701, *p* = 0.005), FF of the knee extensor muscles (quadriceps and sartorius) and knee extension strength (ρ = −0.842, *p* < 0.001), and FF of the knee flexor muscles (hamstring muscles) and knee flexion strength (ρ = −0.864, *p* < 0.001). Fat fraction of the paraspinal muscles also correlated with muscle FF of the thigh muscles and lower leg muscles.

**Conclusion:** In conclusion, patients with Becker muscular dystrophy demonstrate severe paraspinal muscular involvement indicated by low back extension strength and high levels of fat replacement, which parallel involvement of lower limb muscles. Assessment of paraspinal muscle strength and fat replacement may serve as a possible biomarker for both the clinical management and further study of the disease.

## Introduction

Paraspinal muscles consist of erector spinae, which has a superficial segment consisting of the iliocostalis, spinalis, and longissimus muscles, and a deep segment consisting of the multifidus muscle (1). Paraspinal muscles provide trunk stability and mobility and are vital in motor tasks such as rising from a chair and walking. Impairment of these muscles can impact activities of daily living and lead to poor posture, pain, and endurance.

Clinical assessment of paraspinal muscles is challenging because muscle volume is problematic to assess visually and testing the strength of these muscles is inherently difficult. Consequently, the involvement of paraspinal muscles is largely unexplored in patients with a variety of neuromuscular diseases (2). Currently, the prone Biering-Sørensen test is widely used to assess isometric back extension strength. This test uses a hand-held dynamometer which is held over the interscapulum region of the back by the examiner, against which the patient is asked to perform a maximal counter pressure. The test is dependent on the degree of resistance on the hand-held dynamometer generated by the examiner and has been shown to have poor reliability (3) and a ceiling effect as many test individuals overcome the strength of the examiner. An adaptation to the widely used Biodex system to assess muscle strength has introduced a new isometric trunk dynamometer. This system differs from other strength measure tests in that strength is measured from a seated position and is not examiner-dependent. A seated position allows for a feasible measurement of strength, allowing the examiner to assess the strength of weaker patients and removing the challenges of individuals frequently outperforming the examiner's strength.

Magnetic resonance imaging (MRI), using a Dixon technique to assess muscle fat fractions, provides a quantitative method to validate disease distribution and severity of muscle involvement (4–6) and has been shown to correlate with functional assessments and disease progression in several muscular dystrophies (7–12). Previous MRI studies have demonstrated that patients with Becker muscular dystrophy (BMD) exhibit high levels of fat replacement, particularly the hamstrings, adductors, quadriceps, and gastrocnemius muscles (7, 8, 13).

In patients with BMD, a systematic investigation of the paraspinal muscles has not been performed yet. Using a combination of strength measures and quantitative MRI (qMRI), we investigated paraspinal muscle involvement in patients with BMD.

## Materials and Methods

### Study Design and Participants

This cross-sectional study was conducted at the Copenhagen Neuromuscular Center at the National University Hospital, Rigshospitalet in Copenhagen, Denmark from April 2018 to June 2020 in accordance with the declaration of Helsinki and was approved by the Danish National Committee on Health Research Ethics (approval number: H-16030358).

Inclusion criteria were (1) 18 years of age or older, because Copenhagen Neuromuscular Center is a center for adult patients, (2) genetically verified BMD, (3) able to comprehend and adhere to participation requirements, and (4) ability to stand with or without an assistive device or support on wall or furniture. Exclusion criteria were (1) conditions of the spine (i.e., severe scoliosis, past spine surgery) which could interfere with the results, and (2) contraindications to MRI.

Fifty-three patients with BMD from our center were potential candidates to participate in this study. Seven patients did not wish to participate for personal reasons, and it was not possible to obtain contact with nine patients. Another 19 patients were excluded due to exclusion criteria, 15 wheel-chair bound, one cognitively impaired, and three with contraindications to MRI. This left 18 patients who agreed to participate (Table 1). Fourteen age- and body mass index (BMI) -matched healthy men (HC) were included in the study for comparison of findings in patients with BMD. Quantitative muscle strength measure and qMRI were completed in a single visit. The participants were asked to refrain from non-habitual physical activity the day before the visit.

**Table 1:** Demographics of the 18 participating patients with Becker muscular dystrophy.

**Subject ID**	**Age**	**BMI**	**First symptom**	**Age at symptom onset**	**Symptoms at visit**	**Pathogenic variant**	**Disease duration**
BMD 1	45	27.6	Difficulty running, stiffness	28	Severe difficulty walking and rising from a chair. Climbing stairs possible with support.	c.676_678del; p.(Lys226del)	17
BMD 2	50	27.5	Stiffness	41	Moderate to severe pain and joint stiffness.	Del26; p.(Val1145_Lys1201del)	9
BMD 3	36	22.6	Pain	10	Mild pain and joint stiffness.	Del45-48; p.(Glu2147_Gln2366del)	26
BMD 4	30	25.8	Fatigue	10	Moderate fatigue and muscle cramps.	c.6912+1G>T; p.(?)^§^	20
BMD 5	37	28.0	Unable to jump	2	Dependent on an electric wheelchair for most functions. Severe difficulty walking and rising from a chair. Climbing stairs not possible.	Del45-48; p.(Glu2147_Gln2366del)	35
BMD 6	32	25.1	Pain, muscle weakness	29	Severe difficulty walking, rising from a chair, and stair climbing. No use of assistive device.	c.1602 G>A; p.(?)^§^	3
BMD 7	27	23.8	Difficulty climbing stairs	24	Moderate difficulty walking, rising from a chair, and climbing stairs. No use of assistive device.	Del45-47; p.(Glu2147_Lys2304del)	3
BMD 8	33	23.6	Muscle cramps	5	Electric wheelchair for most functions. Able to stand for very short duration with support.	Del45-48; p.(Glu2147_Gln2366del)	28
BMD 9	18	29.9	Fatigue	1	Severe fatigue.	c.5632C>T; p.(Gln1878*)	17
BMD 10	38	23.6	Difficulty climbing stairs	22	Severe difficulty walking, rising from a chair, and climbing stairs. Occasionally uses cane for walking.	Del45-48; p.(Glu2147_Gln2366del)	16
BMD 11	38	25.6	Pain	6	Severe difficulty walking, rising from a chair, and climbing stairs. No use of assistive device	Del45-48; p.(Glu2147_Gln2366del)	32
BMD 12	29	27.4	Asymptomatic	N/A	N/A	Del45-47; p.(Glu2147_Lys2304del)	N/A
BMD 13	31	34.3	Muscle weakness, cramps	8	Severe difficulty walking, rising from a chair, and climbing stairs. No use of assistive device.	Del45-47; p.(Glu2147_Lys2304del)	23
BMD 14	25	24.8	Fatigue	6	Severe difficulty walking, rising from a chair, and climbing stairs. No use of assistive device.	Del45-49; p.(Glu2147_Lys2400del)	19
BMD 15	59	22.9	Difficulty climbing stairs	35	Severe difficulty walking, rising from a chair, and climbing stairs. No use of assistive device.	Del48; p.(Val2305_Gln2366del)	24
BMD 16	59	48.2	Pain, difficulty running	7	Electric wheelchair for most functions. Able to stand for very short duration with support.	Del45-47; p.(Glu2147_Lys2304del)	52
BMD 17	45	34.1	Pain, cramps	17	Pain and severe fatigue.	Del2; p.(Tyr11Phefs*7)	28
BMD 18	18	20.5	Difficulty with activities	4	Severe difficulty walking, rising from a chair, and climbing stairs. No use of assistive device.	Del44; p.(Arg2098Asnfs*16)	14

*Table displays age, BMI, first symptom, age at symptom onset, age at diagnosis, symptoms at visit, pathogenic variant, and disease duration in years. BMI, body-mass-index*.

§ *The variant c.1602G>A predicts a synonymous effect on the protein (p.(Lys534=)), but changes the last base in exon 13, and is predicted to disrupt the 5′ spice site for which the protein consequence cannot be predicted confidently (p.(?))*.

Before data collection, written informed consent was obtained from all participants. Written informed consent was also obtained from the participants for the publication of any potentially identifiable images or data included in this article.

### Quantitative Muscle Strength Measure

The maximal voluntary isometric contraction was measured in the following order: (1) hip flexion and extension in supine position of the dominant leg, (2) knee extension and flexion in sitting position of the dominant leg, and (3) trunk extension and flexion in sitting position. Hip and thigh muscle strength was acquired using a stationary dynamometer (Biodex System 4 Pro, Biodex Medical Systems, Shirley, NY).

Trunk muscle strength was acquired using a Biodex Dual-position back Extension/Flexion attachment (model number 830-450). The anterior superior iliac spine was aligned with the attachment's fixed axis of rotation and back support was set at 100° hip angle. To minimize the influence of muscles from other parts of the body, the chest, and thighs were immobilized with Velcro straps, and the participants were asked to cross their arms in front of their chest during the test. Each participant was instructed to perform a maximal isometric trunk extension and trunk flexion. One contraction lasted 5 s. To ensure maximal contraction, two sub-maximal test trials were performed to familiarize the participants with the testing protocol for each position, followed by three trials of maximal contractions (interchangeably between extension and flexion) with 30 s of rest in between each contraction. Standardized verbal encouragement was provided to each participant during testing. To avoid muscle fatigue, each participant rested for 30 min between the lower limb muscle strength test and trunk muscle strength test.

### MRI Data Acquisition

Images were acquired with a Siemens 3.0 Tesla Magnetom Verio scanner (Erlangen, Germany) at the Department of Radiology at the National University Hospital, Rigshospitalet in Copenhagen, Denmark. Each participant was examined in the headfirst supine position. The MRI protocol was composed of a three-plane localizer sequence followed by a 2-point Dixon sequence (Field of view (FOV) 400–450 mm; slice thickness 3.5 mm; distance factor 0%; repetition time (TR)/echo time (TE) 2.45 and 3.675/5.59 ms; flip angle of 9 deg.) and a T1-weighted sequence (FOV 400–450 mm; slice thickness 6.0 mm; distance factor 20%; TE/TR 19/650 ms; flip angle of 120°).

The 2-point Dixon sequence had an in-plane resolution of 2.1 × 4 × 3.5 and consisted of a multi-slice sequence consisting of 60 slices per sequence. Also, for the Dixon sequence, we used a low flip angle of 9 deg. to suppress T1 relaxation effects to avoid an overestimation of the fat contribution. The T1-weighted sequence was performed to obtain a visual representation of the muscles and was not included in the statistical analysis.

Two body matrix coils and a peripheral leg coil were used for signal detection. The total scan time was approximately 40 min.

### MRI Data Processing

**Figure 1:**
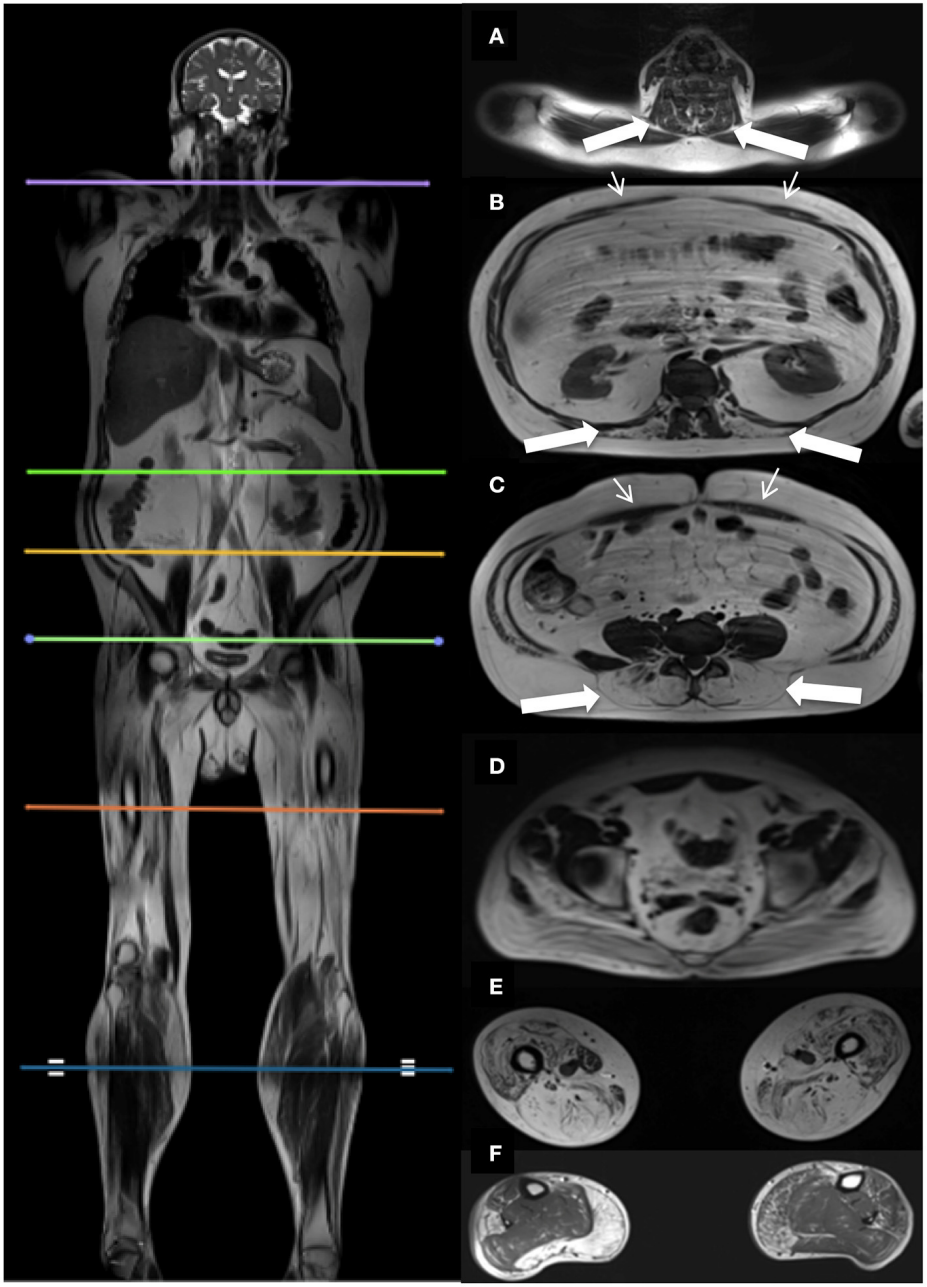
Localizers for cross-sectional MR assessments, and corresponding cross-sectional images. Picture on the left shows the six cross-sectional slices at spinal level C6 **(A)**, Th12 **(B)**, L4/L5 **(C)**, S4 **(D)**, thighs **(E)**, and lower legs **(F)**. Images on the right show the corresponding cross-sectional images. Thick white arrows indicate the paraspinal muscles examined at spinal level C6 **(A)**, Th12 **(B)**, L4/L5 **(C)**. Thin white arrows indicate the abdominal muscles **(B,C)**.

Dixon sequences were used to quantify muscle fat fraction (FF) by defining a region of interest (ROI). Six cross-sectional slices were chosen for the investigation of muscle involvement (Figure 1). Horos software v. 3.3.6 was used to extract information from the 23 bilateral ROIs based on the raw Siemens data. The ROIs were drawn of the in-phase images and fat images and included the following muscles: Erector spinae at spinal level C6, Th12, and L4/L5, and multifidus at spinal level Th12 and L4/L5. Abdominal muscles were not mapped but instead inspected visually due to motion artifacts from respiration. Iliopsoas was mapped at spinal level L4/L5 and gluteus maximus at spinal level S4. Thigh muscles were mapped at mid-thigh, corresponding to 50% of the length of the femur, and included the following muscles: Rectus femoris (RF), vastus lateralis (VL), vastus medialis (VM), vastus intermedius (VI), sartorius (SA), biceps femoris (BF), semitendinosus (ST), semimembranosus (SM), adductor muscles (AM), and gracilis (GR). At the widest section of the lower leg, corresponding to around 1/3 of tibia from the knee down, the following muscles were mapped: tibialis anterior (TA), peroneals (PER), tibialis posterior (TP), soleus (SOL), gastrocnemius lateralis (GL), and gastrocnemius medialis (GM).

We investigated the erector spinae muscle and multifidus muscle at three spinal levels to cover the important segments of the spine following previous MRI studies done in neuromuscular disorders (14–16). The thigh and lower leg muscles were also chosen based on earlier MRI studies (7, 8, 10–13, 17, 18).

Cross-sectional area (CSA) was determined from each ROI followed by a quantitative FF estimation. Mean muscle FF was expressed as


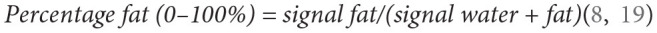


Bilateral mean muscle FF was used in the analysis.

Contractile cross-sectional area (CCSA) was expressed as


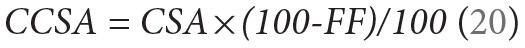


Muscle FF distribution was examined in the thigh and lower leg muscles by examining additional ROIs located 15 slices proximally and 15 slices distally from the center slice of the thigh and lower leg.

### Statistical Analysis

Statistical analysis was performed using SPSS v22. The Mann-Whitney *U*-test was used to test the null hypothesis of no difference in paraspinal muscle FF between BMD and HC and the level of significance was set at *p* ≤ 0.05.

The Friedman test was used to test for differences in FF distribution in the thigh and lower leg muscles. Wilcoxon test was performed with a Bonferroni adjustment on significant differences in FF distribution and the level of significance was set at *p* ≤ 0.017. We report median values and interquartile ranges (Q1, Q3) unless otherwise stated.

Spearman correlation was used to evaluate the correlation between (1) trunk extension strength and paraspinal muscle FF in BMD, (2) hip strength and hip muscle FF in BMD, (3) thigh strength and thigh muscle FF in BMD, (4) paraspinal muscle FF and disease duration, where disease duration was defined as the number of years from symptom onset to the participant's age on the day of the visit, (5) paraspinal muscle FF and age in BMD and HC, (6) paraspinal muscle FF and muscle FF of the thigh muscles and lower leg muscles in BMD, and (7) contractile cross-sectional area and hip flexion strength of iliopsoas muscle, knee extension strength of knee extensor muscles (quadriceps and sartorius), and knee flexion strength of knee flexor muscles (hamstrings) in BMD and HC.

A global muscle FF was defined as the average of all ROIs of one cross-sectional slice and included the following: (1) Th12 (multifidus and erector spinae), (2) L4/L5 (multifidus and erector spinae, not including iliopsoas), (3) combined Th12 and L4/L5 (multifidus and erector spinae), (4) thigh muscles (RF, VL, VM, VI, SA, GR, BF, ST, SM, AM, GR), and (5) lower leg muscles (TA, PER, TP, SOL, GL, GM).

## Results

Becker muscular dystrophy participants had a mean age of 36.1 ± 11.9 years and HC participants were 36.4 ± 11.8 years. The mean BMI of BMD participants was 27.5 ± 6.3 and HC had a BMI of 26.6 ± 2.9.

### Fat Fraction in BMD vs. HC

**Figure 2:**
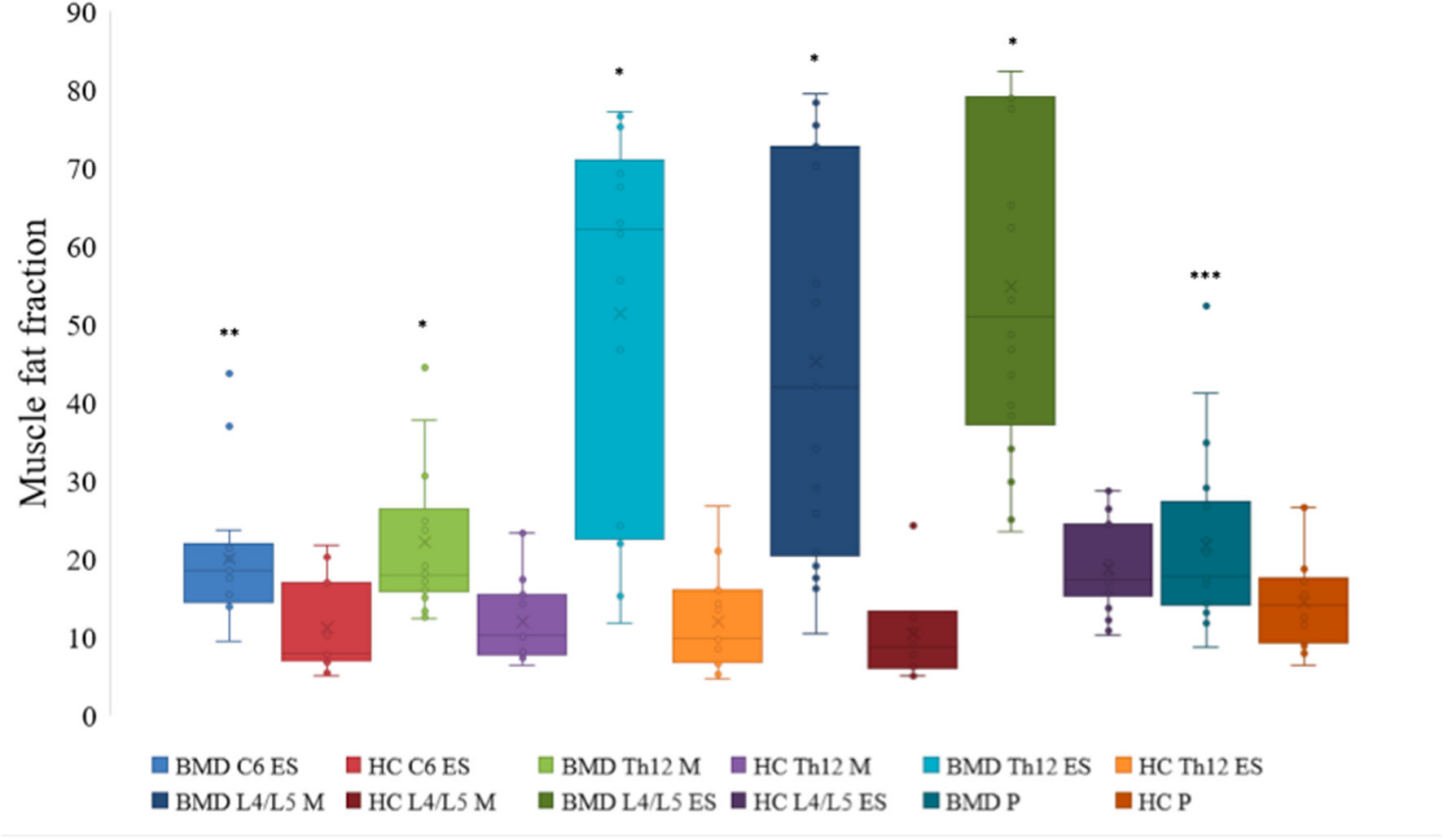
Fat fraction of muscles at spinal level C6, Th12, and L4/L5 in Becker muscular dystrophy and healthy controls. Fat fraction of erector spinae at spinal level C6, Th12, and L4/L5, multifidus at spinal level Th12 and L4/L5, and iliopsoas of participants with Becker muscular dystrophy and healthy controls. Asterix indicate difference from healthy controls *p < 0.001, **p = 0.001, and ***p = 0.023. Missing value for C6 erector spinae (*n* = 1, due to phase-shift artifacts). BMD, Becker muscular dystrophy; HC, Healthy controls; ES, erector spinae; M, multifidus; P, iliopsoas.

All examined paraspinal muscles exhibited severe muscle affection in BMD. When compared to HC, we found a statistically significant difference in median muscle FF on all three spinal levels (Figure 2). The median muscle FF at spinal level C6 erector spinae in participants with BMD was 18.4% (14.5, 21.9%) and in HC FF was 7.9% (6.8, 16.9%). At spinal level Th12, fat replacement of multifidus was 17.8% (15.8, 26.3%), and in erector spinae 62.2% (22.4, 71.1%), and in HC FF was 10.2% (7.7, 15.4%), and 9.7% (6.6, 15.9%), respectively. In the lumbar region, median FF was 41.9% (20.3, 72.7%) in multifidus and 50.9% (37.2, 78.9%) in erector spinae and 9.1% (5.8, 13.2%) and 18.8% (15.7, 26.9%) in HC, respectively. The median muscle FF of iliopsoas in BMD and HC was 17.7% (13.9, 27.3%) and 14.1% (9.2, 17.5%). Furthermore, a visual inspection of the abdominal muscles revealed that the muscles were well-preserved. In addition, MR images obtained on the asymptomatic participant showed modest intramuscular changes in erector spinae and multifidus at spinal level L4/L5.

We found no significant difference in muscle FF distribution of the hamstring muscles, tibialis anterior muscle, and peroneal muscles (*p* > 0.05). There was a significant difference in muscle FF distribution of the quadriceps muscle (x^2^2 = 10.706, *p* = 0.005) and calf muscle (x^2^2 = 13.059, *p* = 0.001). Median muscle FF of the three cross-sectional slices of the quadriceps were as follows: proximal slice 47.7% (15.8, 68%), center slice 52% (19.1, 70.2%), and distal slice 57.7% (22.4, 71.5%). There was a significant difference between the center slice and proximal slice (Z = −2.485, *p* = 0.013) and between the proximal slice and distal slice (Z = −2.595, *p* = 0.003), but there was no significant difference between the center slice and the distal slice (Z = −0.923, *p* = 0.356). Median muscle FF of the three cross-sectional slices of the calf muscle were as follows: proximal slice 18.2% (10.1, 43.3%), center slice 29.1% (14.9, 53.6%), and distal slice 27.7% (11.5, 52.3%). There was a significant difference between the center slice and proximal slice (Z = −3.574, *p* < 0.001) and between the proximal slice and distal slice (Z = −2.722, *p* = 0.006), but there was no significant difference between the center slice and the distal slice (Z = −1.728, *p* = 0.084).

### Fat Fraction and Muscle Strength

**Figure 3:**
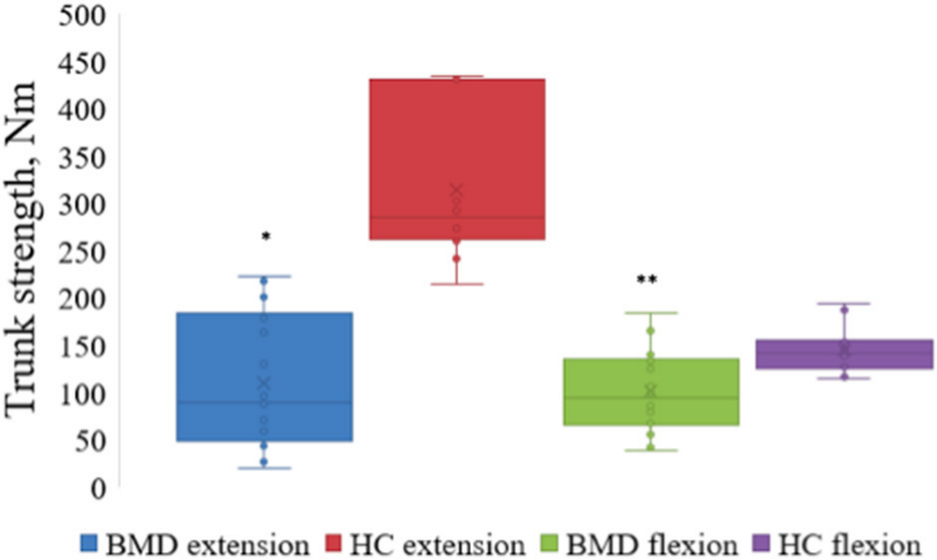
Maximal strength of trunk extension and flexion in Becker muscular dystrophy and healthy controls. Maximal strength of trunk extension and flexion in participants with Becker muscular dystrophy and healthy controls. Asterix indicate difference from healthy controls *p < 0.001, **p = 0.002. Nm, Newton-Meter; BMD, Becker muscular dystrophy; HC, Healthy controls.

**Figure 4:**
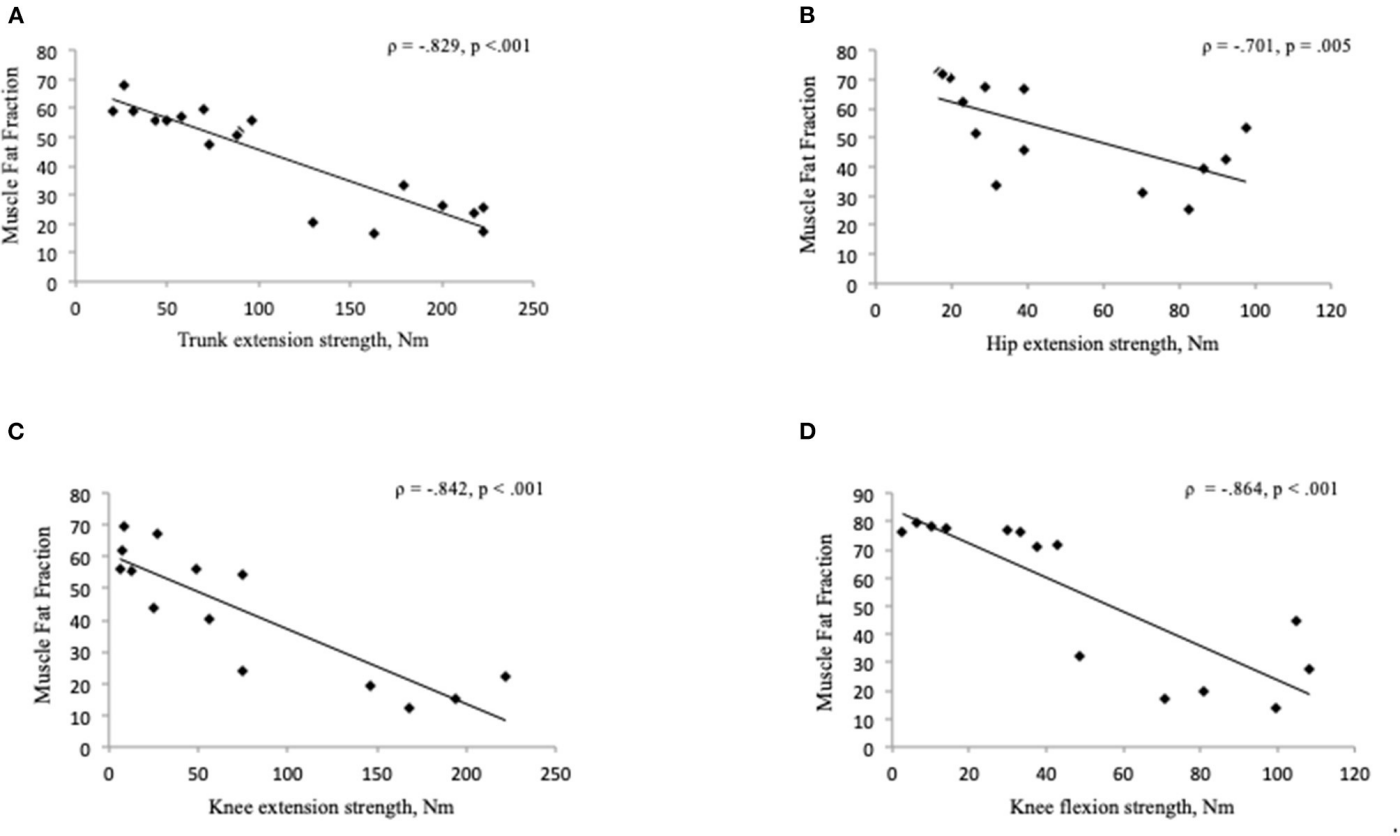
**(A–D)** Correlation between muscle fat fraction and muscle strength in Becker muscular dystrophy. Correlation between global muscle fat fraction of combined Th12 and L4/L5 muscles (multifidus and erector spinae) and maximal trunk extension strength **(A)**, gluteus maximus muscle fat fraction and hip extension strength **(B)**, muscle fat fraction of the knee extensor muscles (quadriceps and sartorius) and knee extension strength **(C)**, muscle fat fraction of the knee flexor muscles (hamstrings) and knee flexion strength **(D)**. Missing value for hip and thigh strength measure (*n* = 4), due to time constraints (*n* = 3), difficulty with positioning in the Biodex chair (*n* = 1), and missing value for muscle fat fraction (*n* = 4), four data sets not used for hip and thigh because hip and thigh strength not measured on four participants. Nm, Newton-Meter.

Participants with BMD had statistically significant lower strength in trunk extension and flexion compared to HC (Figure 3). Median value of maximal trunk extension strength in participants with BMD was 88.4 Nm (48.1, 184.4 Nm) and 284.6 Nm (260.9, 431 Nm) in HC. Median value of trunk flexion strength was 93.7 Nm (65.8, 134.1 Nm) in BMD and 141.1 Nm (124.3, 154.8 Nm) in HC. There was a strong and inverse correlation between global muscle FF of combined Th12 and L4/L5 spinal level muscles (multifidus and erector spinae) and trunk extension strength in participants with BMD (ρ = −0.829, *p* < 0.001) (Figure 4A). The asymptomatic participant displayed a strength of 163.1 Nm in trunk extension and 41.2 Nm in trunk flexion, and his matched healthy control displayed 434.7 Nm in extension and 146.6 in flexion.

Of the 18 BMD participants, 14 also underwent muscle strength measures of the hip and thigh. We found a strong and inverse correlation between gluteus maximus muscle FF and hip extension strength (ρ = −0.701, *p* = 0.005) (Figure 4B) but no significant relationship was found between FF of the iliopsoas muscle and hip flexion strength (ρ = −0.279, *p* = 0.334). A strong and inverse correlation was found between muscle FF of the knee extensor muscles (quadriceps and sartorius) and knee extension strength (ρ = −0.842, *p* < 0.001), and knee flexor muscles (hamstrings) and knee flexion strength (ρ = −0.864, *p* < 0.001) (Figures 4C,D).

### Correlations

**Figure 5:**
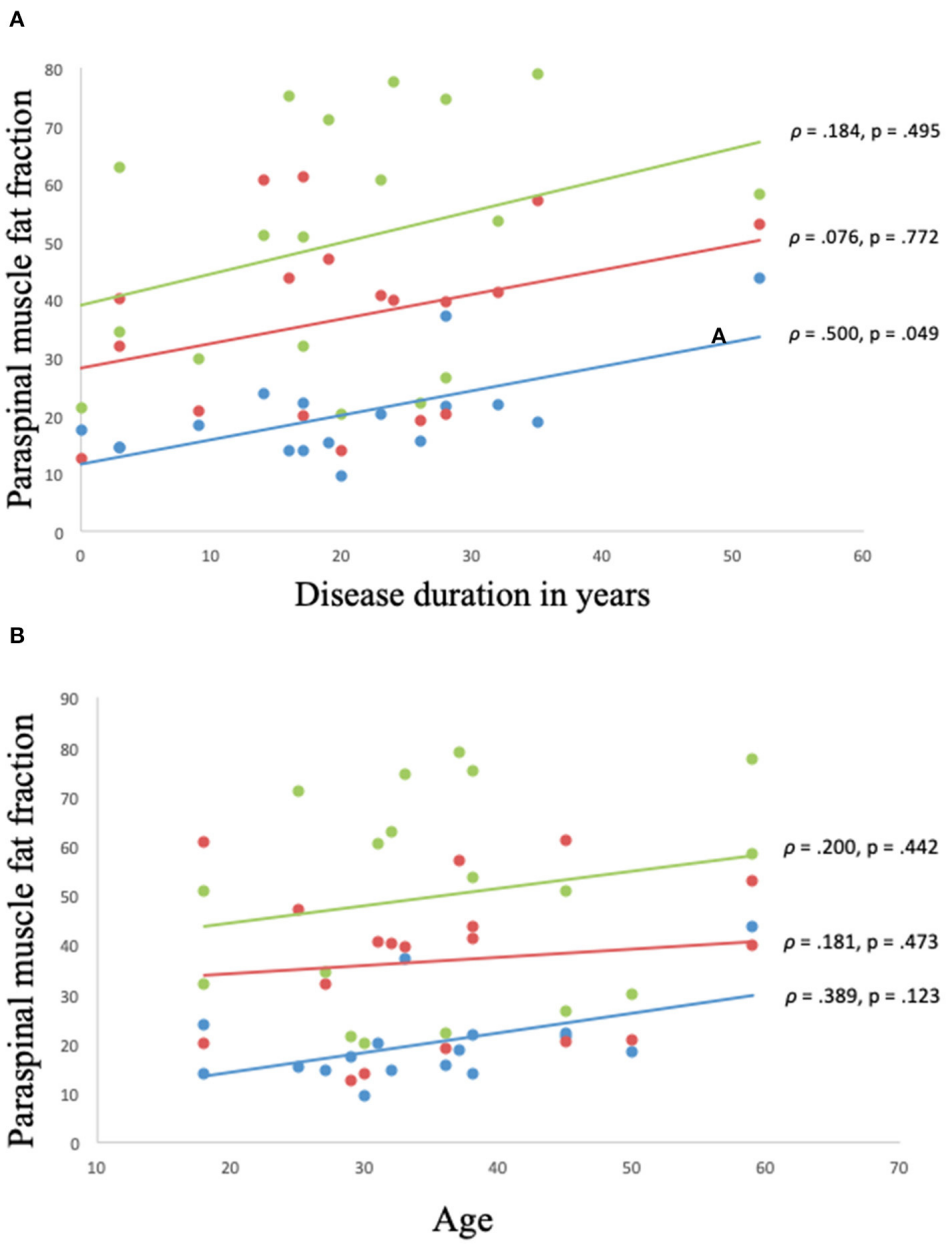
**(A,B)** Correlation between muscle fat fraction of paraspinal muscles and disease duration and age in Becker muscular dystrophy. Correlation between muscle fat fraction and disease duration in Becker muscular dystrophy: Erector spinae fat fraction at C6 spinal level (blue), global muscle fat fraction at spinal level Th12 (red), and global muscle fat fraction at spinal level L4/L5 (green) **(A)**. Correlation between muscle fat fraction and age in Becker muscular dystrophy: Erector spinae fat fraction at C6 spinal level (blue), global muscle fat fraction at spinal Th12 (red), and global muscle fat fraction at spinal level L4/L5 (green) **(B)**. Missing value for **(A,B)** (*n* = 1, due to phase-shift artifacts).

The disease duration was 20.3 ± 12.7 years. There was a moderate correlation between disease duration and muscle FF of C6 erector spinae (ρ = 0.500, *p* = 0.049), but no correlation was found between disease duration and global muscle FF of multifidus and erector spinae at spinal level Th12 and L4/L5 (Figure 5A).

No correlation was found between age and muscle FF of C6 erector spinae, Th12 multifidus and erector spinae, and L4/L5 multifidus and erector spinae (*p* > 0.05) (Figure 5B).

We found a moderate correlation between muscle FF of C6 erector spinae and global muscle FF of the thigh muscles (ρ = 0.568, *p* = 0.022) and global muscle FF of the lower leg muscles (ρ = 0.506, *p* = 0.046). A strong correlation was found between global muscle FF of Th12 multifidus and erector spinae and global muscle FF of the thigh muscles (ρ = 0.713, *p* = 0.001), while the correlation was moderate between global muscle of Th12 multifidus and erector spinae and global muscle FF of the lower leg muscles (ρ = 0.664, *p* = 0.004). There was a strong correlation between global muscle FF of L4/L5 multifidus and erector spinae and global muscle FF of the thigh muscles (ρ = 0.848, *p* < 0.001) and global muscle FF of the lower leg muscles (ρ = 0.725, *p* = 0.001).

**Figure 6:**
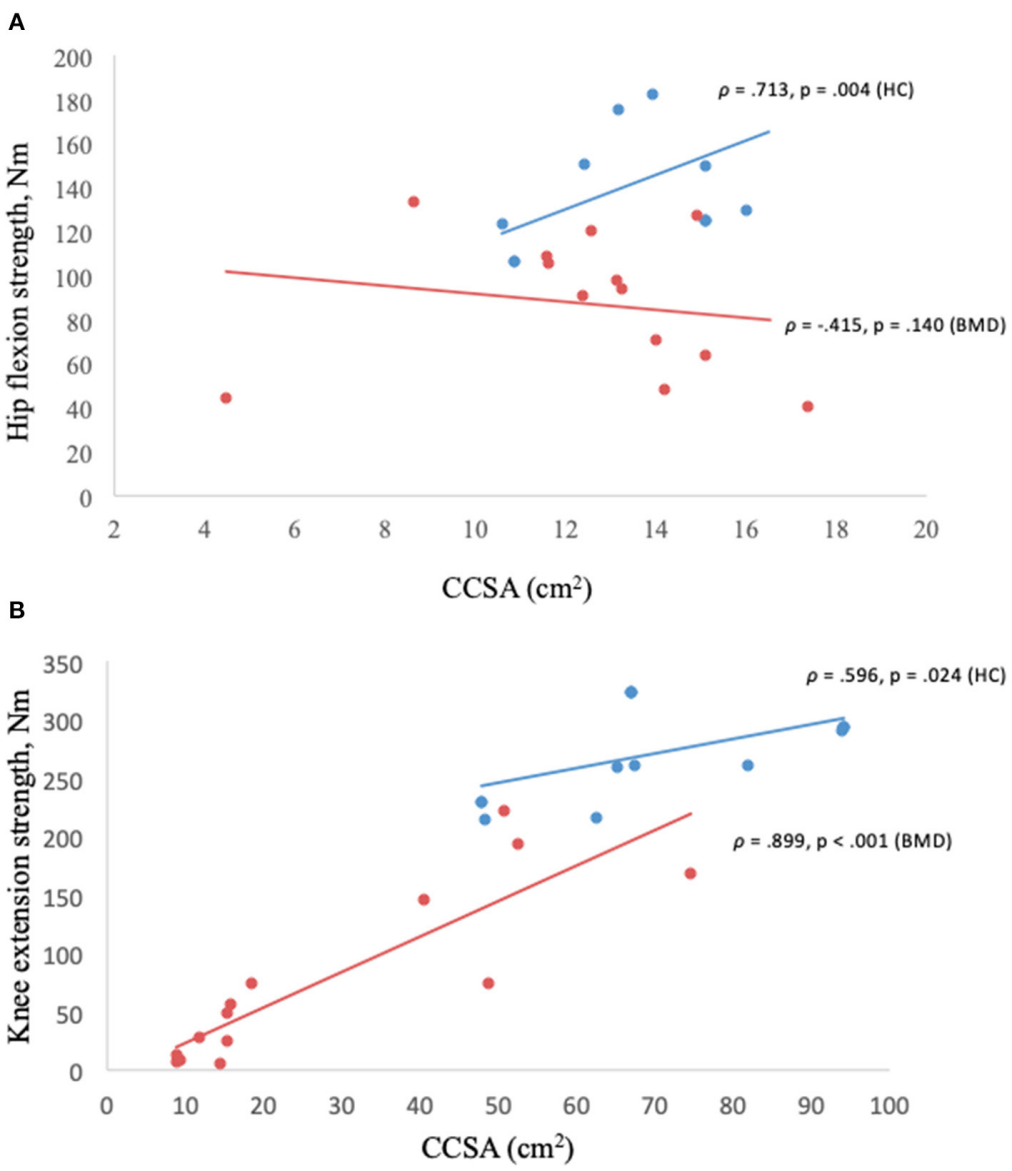
**(A,B)** Muscle strength in relation to contractile cross-sectional area. Hip flexion strength and contractile cross-sectional area of iliopsoas muscle in Becker muscular dystrophy and healthy controls **(A)**, knee extension strength and contractile cross-sectional area of knee extensors (quadriceps and sartorius) in Becker muscular dystrophy and healthy controls **(B)**. Missing value for Becker muscular dystrophy (*n* = 3) due to participant time constraints and (*n* = 1) due to positioning difficulty in the scanner, missing value for healthy controls (*n* = 4) to match sample size of Becker muscular dystrophy participants. BMD, Becker muscular dystrophy; HC, Healthy controls; CCSA, contractile cross-sectional area; Nm, Newton-meter.

Contractile cross-sectional area of the iliopsoas muscle in relation to hip flexion strength lacked correlation (ρ = −0.415, *p* = 0.14) (Figure 6A), but not in the knee extensors (quadriceps and sartorius muscles) (Figure 6B) and knee flexors (hamstrings muscles) (*p* < 0.05).

**Figure 7:**
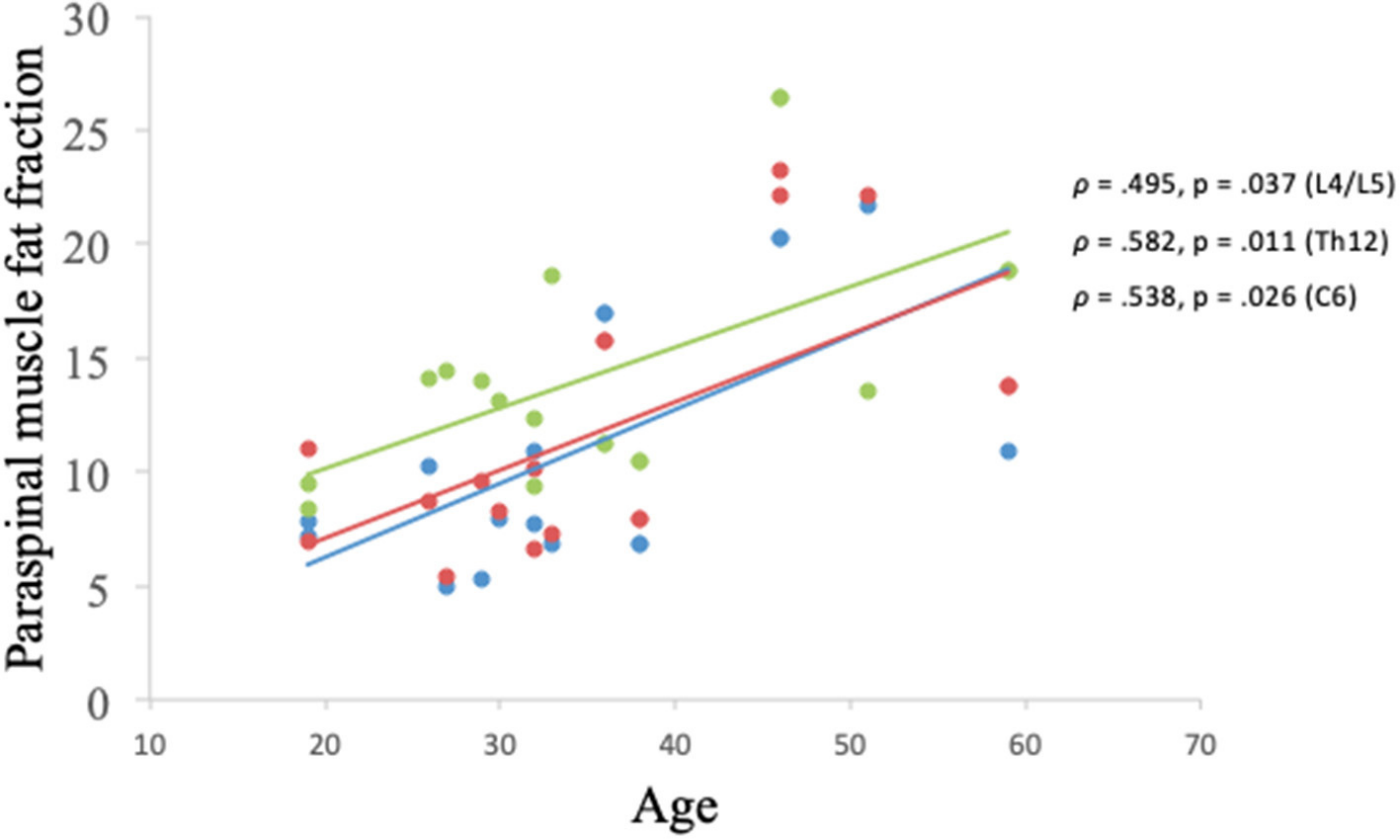
Correlation between muscle fat fraction of paraspinal muscles and age in healthy controls. Erector spinae muscle fat fraction at C6 spinal level (blue), global muscle fat fraction at spinal level Th12 (red), and global muscle fat fraction at spinal level L4/L5 (green).

There was a significant correlation between the age of the HC participants and the muscle FF levels found at spinal level C6, Th12, and L4/L5 (*p* < 0.05) (Figure 7).

## Discussion

This cross-sectional study is the first to systematically examine paraspinal muscle involvement in BMD. We studied the paraspinal muscles in a group of 18 patients with BMD using quantitative measures of trunk muscle strength and qMRI.

The main findings of our study are that paraspinal muscle FF and trunk extension strength differ significantly in patients with BMD compared to HC. Muscle FF of the paraspinal muscles is strongly and inversely correlated with trunk extension strength in BMD and muscle FF of gluteus maximus and thigh muscles correlated strongly and inversely with hip and thigh strength. Additionally, a correlation was found between muscle FF of the paraspinal muscles and muscle involvement of the thigh and lower leg muscles, indicating that disease progression of paraspinal muscles may follow general disease progression in BMD and possibly be considered as a potential biomarker.

Previous MRI studies have discovered paraspinal muscle involvement in other neuromuscular disorders. Dahlqvist et al. (14) reported paraspinal muscle affection in Facioscapulohumeral muscular dystrophy where the authors found a muscle FF of 30% at the cervical spinal level erector spinae, 45% at thoracic spinal level erector spinae, and 40% at the lumbar spinal level erector spinae. In our cohort, we found a lesser muscle affection in erector spinae at the cervical region and a larger affection in multifidus and erector spinae at the thoracic and lumbar region in comparison with Facioscapulohumeral muscular dystrophy. Furthermore, a case study reported paraspinal muscle affection in a patient with McArdle disease (21), and paraspinal muscle involvement was reported in Myotonic muscular dystrophy with large fat replacement observed in erector spinae at the lumbar spinal level (22). Muscle FF of 49.9% was observed in erector spinae at the lumbar spinal level in Duchenne muscular dystrophy (23), and Schreckenbach et al. (24) found paraspinal muscle involvement in two individuals with reducing body myopathy. Frequent involvement of the paraspinal muscles was also found in female carriers of dystrophinopathy (25), underlining the importance of MRI to identify muscle pathology in muscle diseases.

Paraspinal muscles are vital for the stabilization and mobilization of the trunk and consequently, weakness, and impairment of these muscles can have a profound impact on activities of daily living, resulting in a backward bent posture, poor mobility, postural control difficulties, and potential spinal malalignment. We assessed trunk strength using Biodex Dual-position back Extension/Flexion attachment unit. Biodex is a consistent method to assess muscle strength and has shown to be a reliable method to measure trunk muscle strength (26). Muscle FF found in the paraspinal muscles was strongly and inversely associated with reduced muscle strength in trunk extension in participants with BMD. A similar association between trunk extension strength and muscle FF was demonstrated by Dahlqvist et al. (14) where the authors found a connection between paraspinal muscle FF and back extension strength in Facioscapulohumeral muscular dystrophy. Furthermore, Schlaeger et al. (27) observed a relationship between muscle FF of erector spinae and back extension strength in healthy participants and found a muscle FF of 9%. In our study, the healthy population had a muscle FF of about 20%. The higher level of FF in our HC participants could be explained by age, in that age ranged between 18 and 59 years and it has previously been shown that paraspinal muscles are more susceptible to fat infiltration with aging (15), and Hadar et al. (28) also found a higher level of fat replacement in older males. Additionally, age in our HC group was related to muscle FF of the paraspinal muscles, despite a small sample size (Figure 7). In addition, Schlaeger et al. combined erector spinae and multifidus when determining the cross-sectional area, whereas, we separated the two muscles in our study. However, a combined muscle FF of erector spinae and multifidus resulted in a muscle FF of about 15% in our HC participants. Furthermore, Dahlqvist et al. (14) found an FF of 20% in their healthy population, which corresponds well with our study. Moreover, a previous study found a connection between fat replacement in the calf muscle and weakness in plantar flexors in patients with BMD, amplifying the relationship between muscle FF and muscle strength (20).

There was no correlation between disease duration and muscle FF of the paraspinal muscles, except at spinal level C6 erector spinae, and age, which could be explained by the heterogeneity of the clinical phenotype of BMD. In addition, we found a difference in muscle FF distribution in the quadriceps muscle and calf muscle, suggesting that muscle FF distribution is not homogenous in these muscles. Furthermore, muscle involvement is less severe in the proximal part of the quadriceps and calf muscles in relation to the center and distal slice of the muscles.

We found a large muscle FF in the gluteus maximus muscle, corresponding well to previous studies where a severe involvement of the gluteus maximus in BMD was established (13, 17, 29). The iliopsoas muscle was less affected in participants with BMD relative to the muscles of the thoracic spine, lumbar spine, and lower limbs. This finding agrees with an earlier study where the authors found that iliopsoas was less affected in patients with BMD relative to lower limb muscles (13). In other neuromuscular disorders, however, iliopsoas is prominently affected, such as in Bethlem myopathy (30), Hypokalemic Periodic Paralysis (31), and Limb-Girdle muscular dystrophy type R9 (32). The difference in the level of muscle affection in iliopsoas and paraspinal muscles may be linked to the specific molecular defect, but also variability in muscle fiber composition of these muscles. Earlier studies have shown that iliopsoas has a predominance of type 2A muscle fibers (33) and paraspinal muscles have a predominance of type 1 fibers (34). However, whether muscle fiber composition has an impact on the level of muscle involvement in BMD is unknown. Furthermore, our study showed that contractile properties of the iliopsoas muscle in relation to hip flexion strength were disrupted. A previous study also reported a contractile disruption of the calf muscle in BMD (20). In Duchenne muscular dystrophy, Wokke et al. (35) found a correlation between CSAA and muscle strength in the quadriceps muscle and hamstring muscles which corresponds well with our findings in the quadriceps and hamstring muscles in BMD. Additionally, Wokke et al. (35) found a disruption in contractile properties in the triceps surae muscle, resembling the findings of Løkken et al. (20). This suggests that there may be similarities to be found in the contractile properties in BMD and Duchenne muscular dystrophy. Disrupted contractile properties have also been found in other muscle disorders such as congenital myopathy (36) and spinobulbar muscular atrophy (37). Knee extension muscle strength ranged between 6.1 and 222 Nm in BMD and in HC the strength ranged between 214.8 and 323.9 Nm, indicating that patients with BMD have about 31–97% reduced muscle strength than HC. Despite a substantially lower muscle strength in knee extension, contractile properties in the quadriceps and sartorius muscles were not disrupted in BMD, suggesting that contractile properties may be related to muscle type, muscle fiber type composition, and the level of severity of muscle involvement.

Imaging studies using visual rating scales in BMD have distinctively been able to show patterns of muscle involvement (13, 29). However, to quantify pathological changes in the muscle and to monitor disease progression, qMRI has been shown to be more sensitive than a visual rating scale (8, 19). Quantitative MRI represents a valid biomarker and provides an objective endpoint to measure disease distribution (38). Intramuscular changes observed in the asymptomatic BMD participant show that MRI can detect subtle intramuscular degenerative changes before symptoms occur. Furthermore, previous studies done in patients with BMD have presented a correlation between muscle FF in the lower limbs and functional measures such as 6-min walk test, motor function measure, and the north star ambulatory assessment (7, 8, 19), suggesting that qMRI is valuable in the evaluation of the disease as a complementary tool to clinical functional testing.

Our study has some limitations: The investigated cohort of patients is relatively small. However, we were able to determine a relatively consistent muscle involvement in the paraspinal muscles. We did not test isokinetic muscle strength because the weight of the trunk flexion and extension unit exceeded what the majority of the patients could manage, and therefore, it is unknown whether isokinetic trunk strength assessment provides similar results. However, isometric testing is a reliable method to identify maximal strength in a muscle and has been shown to have a high intra-class correlation in relation to isokinetic testing (39). A 2-point Dixon technique may be more sensitive to phase-shift artifacts than a 3-point Dixon technique (40); However, we experienced phase-shift in just one participant at spinal level C6 and that one image was not included in the analysis.

We conclude that patients with BMD demonstrate severe paraspinal muscle involvement. The level of muscle involvement is based on fat replacement within the muscle and decreased muscle strength. We found high levels of fat replacement of the paraspinal muscles which strongly and inversely correlates with reduced muscle strength in trunk extension and the level of FF in the paraspinal muscles parallel involvement of the lower limb muscles. Findings from this study contribute to the clinical management of patients with BMD and suggest that assessment of paraspinal muscle strength, and fat replacement may serve as a possible biomarker in longitudinal investigations of BMD.

## Data Availability Statement

The original contributions presented in the study are included in the article/supplementary material, further inquiries can be directed to the corresponding author/s.

## Ethics Statement

The studies involving human participants were reviewed and approved by the Danish National Committee on Health Research Ethics (approval number: H-16030358). The patients/participants provided their written informed consent to participate in this study. Written informed consent was obtained from the participants for the publication of any potentially identifiable images or data included in this article.

## Author Contributions

AMS contributed with data collection, data analysis, and drafting of manuscript. KR contributed with study design, data collection, and drafting of manuscript. JS contributed with data collection. TK contributed with data collection. NW contributed with study design and drafting of manuscript. JV contributed with study design, data analysis, and drafting of manuscript. All authors contributed to the article and approved the submitted version.

## Conflict of Interest

The authors declare that the research was conducted in the absence of any commercial or financial relationships that could be construed as a potential conflict of interest.
